# A New Dental Journal

**Published:** 1881-01

**Authors:** 


### OHIO STATE DENTAL SOCIETY.

### A NEW DENTAL JOURNAL.

The Prospectus of "The Ohio State Journal of Dental Science" has just come to hand. This is to be a bi-monthly journal, edited by Dr. Geo. Watt, of Xenia, O., and published by Ransom & Randolph, of Toledo. The first number will appear about Feb. 15th, and on each alternate month at that date. Dr. Watt's known ability is sufficient guarantee for the character and value of the journal. The improvement of Dr. Watt's health, to the extent of enabling him to undertake such a work, is a matter of great gratification to his friends and to the whole profession. It was feared, for the past few years, that the days of his greatest usefulness were passed; but he seems to have renewed his age and strength, and all will rejoice to learn that he is again to be heard, through the press, at least. His work at the recent meeting of the Ohio State Dental Society was a surprise to his friends, and inspires a hope that for years to come he may be heard in many of our professional gatherings.

